# Supplementary material for: Association between oral microbiome and seven types of cancers in East Asian population: a two-sample Mendelian randomization analysis
Source: Front Mol Biosci. 2023 Nov 21;10:1327893. doi: 10.3389/fmolb.2023.1327893 (PMC10702768; doi:10.3389/fmolb.2023.1327893)
Supplement: Supplementary file 1 [file DataSheet1.ZIP › Supplementary Materials/MR plots of saliva/saliva═╝/ovarian cancer/pheno.150_to_ovarian cancer_leave_one_out.pdf]

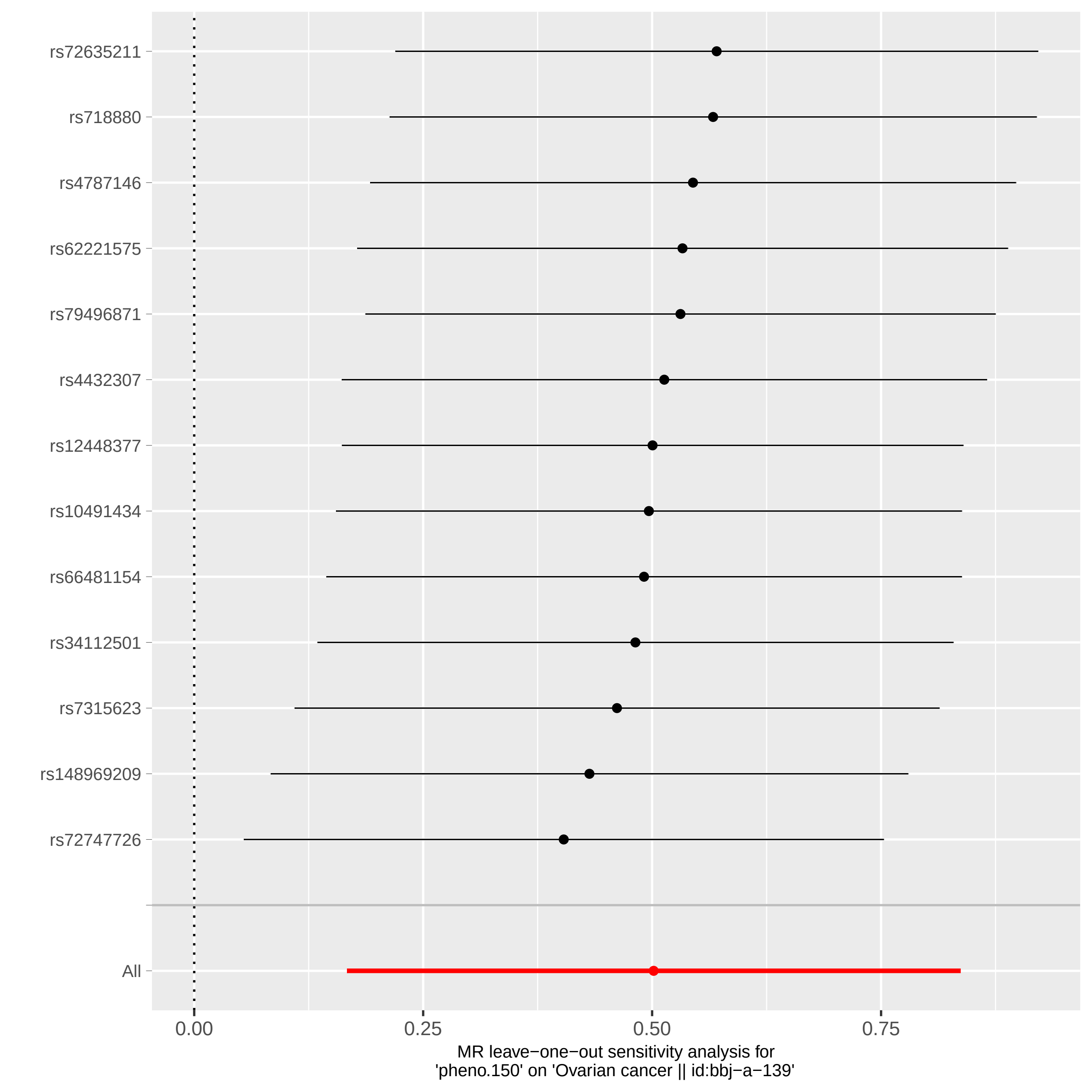
rs72635211
rs718880
rs4787146
rs62221575
rs79496871
rs4432307
rs12448377
rs10491434
rs66481154
rs34112501
rs7315623
rs148969209
rs72747726
All
0.00
0.25
0.50
0.75
MR leave−one−out sensitivity analysis for
'pheno.150' on 'Ovarian cancer || id:bbj−a−139'
